# RAF1 contributes to cell proliferation and STAT3 activation in colorectal cancer independently of microsatellite and KRAS status

**DOI:** 10.1038/s41388-023-02683-w

**Published:** 2023-04-05

**Authors:** Coralie Dorard, Claire Madry, Olivier Buhard, Stefanie Toifl, Sebastian Didusch, Toky Ratovomanana, Quentin Letourneur, Helmut Dolznig, Mathew J. Garnett, Alex Duval, Manuela Baccarini

**Affiliations:** 1 Department of Microbiology, Immunology and Genetics, Center of Molecular Biology, University of Vienna, Max Perutz Labs, Doktor-Bohr-Gasse 9, 1030 Vienna, Austria; 2 Sorbonne Université, INSERM, Unité Mixte de Recherche Scientifique 938 and SIRIC CURAMUS, Centre de Recherche Saint-Antoine (CRSA), Equipe Instabilité des Microsatellites et Cancer, Equipe Labellisée par la Ligue Nationale Contre le Cancer, F-75012 Paris, France; 3 Institute of Medical Genetics, Medical University of Vienna, Waehringer Straße 10, A-1090 Vienna, Austria; 4 Wellcome Sanger Institute, Cambridge, UK

**Keywords:** Colorectal cancer, Cell signalling

## Abstract

More than 30% of all human cancers are driven by RAS mutations and activating KRAS mutations are present in 40% of colorectal cancer (CRC) in the two main CRC subgroups, MSS (Microsatellite Stable) and MSI (Microsatellite Instable). Studies in RAS-driven tumors have shown essential roles of the RAS effectors RAF and specifically of RAF1, which can be dependent or independent of RAF’s ability to activate the MEK/ERK module. In this study, we demonstrate that RAF1, but not its kinase activity, plays a crucial role in the proliferation of both MSI and MSS CRC cell line-derived spheroids and patient-derived organoids, and independently of KRAS mutation status. Moreover, we could define a RAF1 transcriptomic signature which includes genes that contribute to STAT3 activation, and could demonstrate that RAF1 ablation decreases STAT3 phosphorylation in all CRC spheroids tested. The genes involved in STAT3 activation as well as STAT3 targets promoting angiogenesis were also downregulated in human primary tumors expressing low levels of RAF1. These results indicate that RAF1 could be an attractive therapeutic target in both MSI and MSS CRC regardless of their *KRAS* status and support the development of selective RAF1 degraders rather than RAF1 inhibitors for clinical use in combination therapies.

## Introduction

The RAS/RAF/MEK/ERK pathway is a highly conserved signaling cascade that regulates cell morphology, migration, proliferation, differentiation and survival in a tissue and molecular context-dependent manner [1]. Oncogenic mutations in *RAS* drive 30% of all human cancer types [2]. *KRAS*, the most frequently mutated RAS isoform (85%), is found predominantly in pancreatic, lung and colorectal cancer. Promising clinical data are emerging for *KRAS*^*G12C*^ inhibitors [3, 4], but treatment of KRAS-mutated cancer remains challenging. Targeting upstream components of the ERK pathway such as the EGFR or downstream effectors such as MEK is either not recommended for the treatment of KRAS-mutated cancers or not sufficient as single agent [5]. Resistance to targeted therapies might also be due to the complexity of the pathway. There are three *RAF* (*ARAF*, *BRAF* and *RAF1*), two *MEK* (*MEK1* and *MEK2*) and two *ERK* (*ERK1* and *ERK2*) genes encoding for proteins with specific functions. Several studies have demonstrated that RAS-driven cancer are addicted to RAF proteins, but the mechanisms underlying their essential roles are different depending on the cancer type [2]. While RAS-driven melanoma and pancreatic ductal adenocarcinoma are highly dependent on RAF proteins to activate MEK/ERK [6, 7], some other cancers require RAF1-specific functions that are MEK-independent, such as squamous cell carcinoma [8] and lung adenocarcinoma [9–11]. RAF1 can regulate biological processes such as apoptosis through its interaction with ASK1 and MST2 [12, 13], proliferation through PLK1 and CHK2 [14, 15] and migration/differentiation via its interaction with ROKα [16]. Recent studies have highlighted kinase-independent roles of RAF1 in KRAS mutant lung adenocarcinoma that could either be anti-apoptotic [11] or pro-proliferative [17].

Little is known about the mechanistic role of RAF proteins and especially of RAF1 in colorectal cancer (CRC). CRC is the third most common cancer worldwide in terms of incidence and the second in terms of mortality [18]. It is a heterogeneous disease that arises through different mechanisms, with two mutually exclusive subtypes of these tumors reported so far, i.e., CRC displaying microsatellite instability (MSI) or chromosomal instability (MSS for MicroSatellite Stable) [19]. At the molecular level, MSS is frequently associated with inactivating mutations of *APC* (Adenomatous Polyposis Coli), while the MSI phenotype (about 15% of CRC) is due to an accumulation of mutations in the microsatellite sequences caused by a defect in the DNA mismatch repair system [20]. In CRC, KRAS mutations occur in about 40% of the cases and are more prevalent in the MSS than in MSI group which in contrast more frequently display BRAF activating mutations [20]. Understanding signaling and molecular mechanisms downstream of mutated KRAS might help to develop treatments for these tumors that do not respond to therapies such as anti-EGFR [5].

A crucial role of RAF proteins in KRAS-mutated CRC has been postulated previously. On one hand, silencing of RAF and autophagy inhibition leads to cell cycle arrest and cell death in KRAS mutant cells [21], a phenotype similar to the one observed in KRAS lung adenocarcinoma when only RAF1 is genetically ablated [11, 17]. On the other hand, targeting RAF1 with selective inhibitors synergizes with MEK inhibition in MSS CRC cell lines harboring a KRAS mutation [22], with a prominent role of RAF1, as also described in human colonosphere cultures in which genetic depletion of RAF1 impairs clonogenic and tumorigenic properties of CRC cells [23]. Research on RAF1-specific functions focused on its impact in KRAS mutated samples [23] or MSS cell lines [22]. Based on this, we aimed to elucidate whether RAF1 is required as an effector of mutated *KRAS* in MSI and MSS CRC, or whether it might have a role involving other pathways. Here, we demonstrate a crucial role of RAF1 in the proliferation of CRC cell lines both in 2D and 3D cultures. This role is independent of KRAS and MS status and requires an intact dimerization surface but not RAF1 catalytic activity. The decrease in proliferation caused by RAF1 loss is potentiated by MEK inhibition. Loss of RAF1 in CRC spheroids deregulates a group of genes that impact the transcription factor STAT3 and its downstream targets implicated in angiogenesis in human primary CRC. The data indicate that selective degraders of RAF1 might be more effective than selective inhibitors.

## Results

### RAF1 is required for CRC spheroid growth independently of KRAS mutational status, microsatellite phenotype and molecular classification

**Fig. 1:**
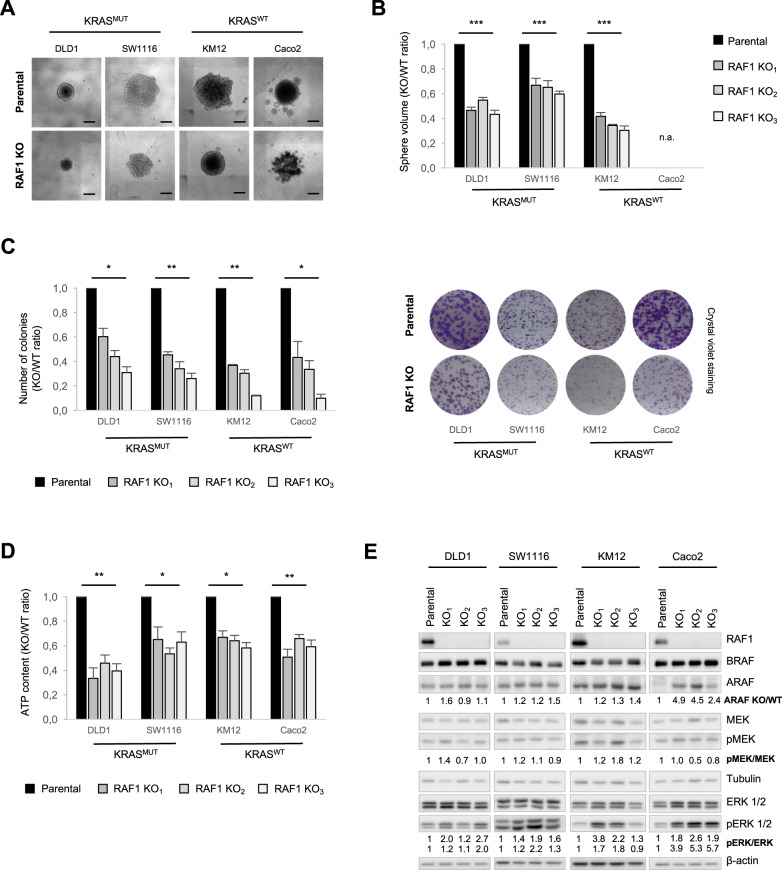
RAF1 loss reduces CRC cell proliferation without affecting ERK activity. **A** Ablation of RAF1 reduces spheroid size 5 days after spheroid formation in four different CRC cell lines. Pictures are representative of three independent RAF1 KO and more than three independent experiments. Scale bars = 500 µm. **B** Spheroid volume was measured 5 days after spheroid formation in three independent RAF1 KO per cell line. The plot shows the ratio between the volume of the KO/WT spheroids. Experiments were performed at least three times. **C** Colony formation assay on cells plated in 2D at low density; colonies were counted 12 days after plating (left panel). Representative pictures of colonies are shown in the right panel. **D** ATP content 5 days after spheroid formation is reduced in three independent RAF1 KO clones/cell line compared to the parental cell lines. **E** Immunoblot showing RAF, MEK and ERK status was performed on 3D spheroids at day 5. The blot is representative of three independent experiments. Numbers represent the ratio KO/WT of the indicated protein after quantification, and Tubulin or β-actin was used as a loading control. **B**–**D**, *p < 0.05, **p < 0.01, ***p < 0.001, n.a not applicable.

To investigate its role in CRC, RAF1 was ablated by CRISPR/cas9 genome editing in four CRC cell lines with different KRAS mutational status, microsatellite (MS) phenotype [24] or consensus molecular subtype (CMS) defined by the CRC Subtyping Consortium [24, 25] (RAF1 knockout, KO; Supplementary Table 1). Spheroid morphology was similar in RAF1-proficient and -deficient cell lines, with the exception of the Caco2 cell line where cells are less compact in the absence of RAF1 (Supplementary Fig. S1A). RAF1 loss significantly reduced proliferation in 3D cultures, as demonstrated by a significant reduction of spheroid volume in three independent RAF1 KO clones of DLD1 (KRAS^MUT^/MSI; CMS1), SW1116 (KRAS^MUT^/MSS; CMS2) and KM12 (KRAS^WT^/MSI; CMS1) cell lines (Fig. 1A, B). In the RAF1-deficient Caco2 (KRAS^WT^/MSS; CMS4) cell line, less cells were observed (Fig. 1A). However, due to its adenomatous phenotype [26], the spheroid volume could not be assessed (Fig. 1B). In line with this, loss of RAF1 also slowed down proliferation in 2D cultures (Supplementary Fig. S1B) and decreased the ability of the cell lines to form colonies (Fig. 1C). We measured ATP content in 3D spheroids lacking RAF1 and demonstrated a general decrease in metabolic activity of RAF1 KO cells (Fig. 1D). The four cell lines described above represent three of the four molecular subtypes defined by the CRC Subtyping Consortium [24, 25]. The missing one (CSM3) is the least frequent and, accordingly, is represented in very few cell lines [24, 25] (Supplementary Table 1). To complement the experiment in Fig. 1, we stably expressed two independent doxycycline-inducible RAF1 shRNA, previously shown to reduce proliferation in DLD1 spheroids (Supplementary Fig. S1C–F), in the LS174T CRC cell line (KRAS^MUT^/MSI; CSM3). Downregulation of RAF1 by two specific shRNA or one siRNA also impacted the size and metabolic activity of spheroids derived from the CMS3 group (Supplementary Fig. S1G–I). Thus RAF1 ablation and, to a lesser extent, downregulation, impacts the proliferation of spheroids derived from 5 CRC cell lines that differ with respect to their KRAS mutation status, MSI/MSS phenotype, and CMS classification (Supplementary Table 2). To gain insight into the proliferation defect caused by RAF1 ablation in the spheroid model, we investigated the activity of the ERK cascade, which is a target of RAF proteins and a master regulator of proliferation [1]. MEK phosphorylation on RAF-dependent sites was not clearly affected by RAF1 ablation, but we observed a constant increase of ERK phosphorylation in all spheroids tested (Fig. 1E), similar to that recently observed in RAF1-deficient lung cancer cell lines [17]. This suggests compensatory functions of other RAF proteins, such as the ones reported in NRAS-mutant melanoma [6], or a more specific function of ARAF, which is able to activate RAS by antagonizing NF1 binding [27]. Neither BRAF nor ARAF expression were significantly changed in RAF1-deficient cell lines, with the exception of the Caco2 cell line in which ARAF was strongly upregulated upon RAF1 loss (Fig. 1E). It is possible that the distinctive ARAF upregulation in RAF1-deficient Caco2 cells might determine their failure to form compact spheroids, developing instead in a multitude of small spheres that do not coalesce. In support of this, ARAF dimers have been found to promote ERK signaling and cell cycle arrest in KRAS-mutated, RAF1-deficient lung adenocarcinoma cell lines [17].

### RAF1 loss delays cell cycle progression

**Fig. 2:**
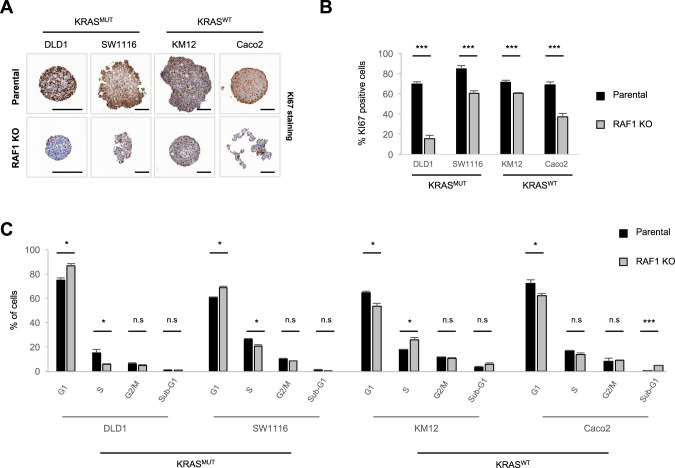
Ablation of RAF1 results in cell cycle defects. **A** KI67 staining of spheroids after 5 days in culture. KO pictures are representative of three independent clones/cell line. Scale bars = 200 µm. **B** Quantification of KI67-positive cells in parental and RAF1 KO spheroids. **C** 5 days after spheroid formation, spheroids were incubated with 10 µM EdU for 2 h, dissociated, fixed and stained. Cell cycle phases were determined using flow cytometry. RAF1 KO plot represents the three independent RAF1 KO clones for each cell line (*n* = 3 separate experiments). **B**, **C**, *p < 0.05, **p < 0.01, ***p < 0.001, n.s not significant.

In line with the observed growth defect, RAF1 KO spheroids displayed a reduced number of proliferating (KI67+) cells (Fig. 2A, B). RAF1 has been already described as an important player in the regulation of cell cycle and mitosis checkpoints [14, 15]. In the 3D spheroid model, we observed cell line-dependent effects of RAF1 ablation on the cell cycle (Fig. 2C). Specifically, the three independent RAF1 KO clones generated in KRAS mutated cells (DLD1 and SW1116) accumulated significantly in G1 phase, while in the context of KRAS WT, RAF1 KO clones (KM12 and Caco2) displayed less cells in G1 phase and were enriched in S or sub-G1 phase. In addition to impacting proliferation, RAF1 could exert anti-apoptotic functions, as observed in lung adenocarcinomas [11, 28]. However, only DLD1 and Caco2 showed increased apoptosis in the absence of RAF1, as determined by Annexin V staining (Supplementary Fig. S2A) and cleaved caspase 3 IHC staining performed on spheroids (Supplementary Fig. S2B, C). Therefore, loss of RAF1 induces both decreased proliferation and increased apoptosis, but neither of these phenotypes is restricted to, or correlates with, a specific KRAS or MSI/MSS status.

### The effects of RAF1 on CRC proliferation are independent of kinase activity and are enhanced by MEK inhibition

**Fig. 3:**
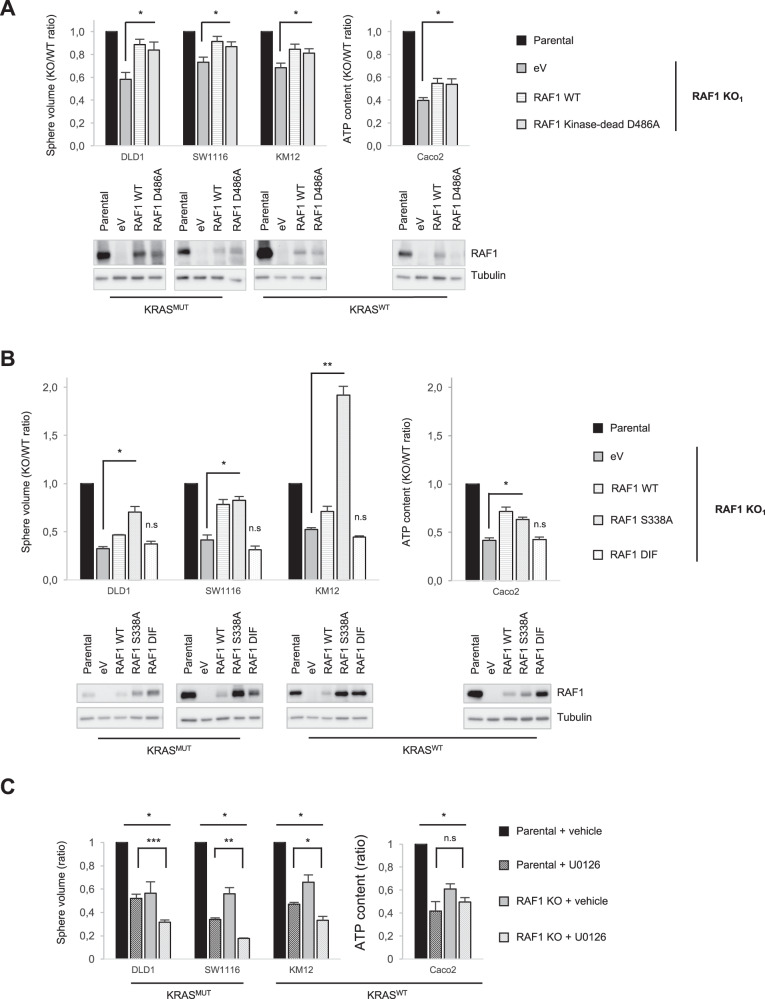
RAF1 kinase activity is dispensable for proliferation while intact dimerization is required, and RAF1 loss combined with MEK inhibition strongly affects CRC cells. **A** RAF1 KO cell lines were stably transfected with either an empty vector (eV), RAF1 full length (WT) or the RAF1 kinase-dead mutant D486A. Spheroid volume or ATP content were determined 5 days after spheroid formation. The parental cell lines were compared with their respective RAF1 KO_1_ clone reconstituted with empty vector or with the RAF1 constructs. The experiment was repeated at least three times (top panel). The Western blot shows RAF1 expression compared to the parental cell lines. Tubulin was used as a loading control (bottom panel). **B** RAF1 KO cell lines were stably transfected with either an empty vector (eV), RAF1 full length (WT), the phosphoablative mutant S338A, or the DIF mutant which unables dimerization. Spheroid volume or ATP content were measured 5 days after spheroid formation. The parental cell lines were compared with their respective RAF1 KO_1_ clone reconstituted with empty vector or RAF1 constructs. The experiment was performed three times (top panel). Immunoblot shows RAF1 expression compared to the parental cell lines. Tubulin was used as a loading control (bottom panel). **C** Parental and RAF1 KO spheroids were treated for 5 days with either DMSO (vehicle), 10 µM U0126 (DLD1, SW116 and Caco2) or 1 µM U0126 (KM12). Spheroid volume or ATP content was determined at the end of the experiment. The plot represents the mean of three independent RAF1 KO clones.The experiment was performed at least three times. **A**–**C**, *p < 0.05, **p < 0.01, ***p < 0.001, n.s not significant.

RAF1 has been shown to have both kinase-dependent and -independent functions. To assess the role of RAF1’s enzymatic activity, we re-expressed RAF1 full length (wild-type, WT) or the kinase-dead mutant D486A, in which aspartic acid residue 486 within the DFG motif of the activation segment was converted to alanine [29], in two independent RAF1 KO clones of each CRC cell line. Both RAF1 WT and the RAF1 kinase-dead mutant increased spheroid volume to a comparable extent, indicating that the proliferation defect is due to RAF1 loss and that RAF1 kinase activity is not required for CRC spheroid proliferation (Fig. 3A, top panel, and Supplementary Fig. S3A). As previously described [16], the expression of RAF1 WT and kinase-dead was weaker than that of endogenous RAF1 in the parental cell lines (Fig. 3A, bottom panel). Accordingly, these constructs increased proliferation but did not bring it back to parental levels. This stoichiometry-dependent effect mirrors the lesser impact on spheroid proliferation achieved by RAF1 downregulation (by sh or siRNA) compared to RAF1 KO (Supplementary Fig. S1C–F and Supplementary Table 2), and suggests a kinase-independent functions of RAF1 [8]. In line with this, the reintroduction of the phosphoablative RAF1 S338A, which cannot be activated by growth factors, restored and in some cases strongly increased spheroid proliferation in all the cell lines tested (Fig. 3B). In contrast, a dimer interface (DIF) mutant containing three mutations (R401H, L407G, and M409W) that prevent RAF dimerization [30] was not able to promote proliferation in RAF1 KO cells (Fig. 3B, top panel), although it was expressed at levels comparable to, or even higher than, endogenous RAF1 (Fig. 3B, bottom panel). Taken together, these results indicate that RAF1 expression and its dimerization interface, but not its kinase activity, are necessary to induce CRC cell proliferation. Confirming these results, treatment with the RAF1-selective kinase inhibitor GW5074 did not impact spheroid volume in RAF1-proficient or -deficient cells (Supplementary Fig. S3B). These results are consistent with recent studies in lung adenocarcinoma demonstrating kinase-independent roles of RAF1 [11, 17]. We then hypothesized that RAF1-deficient CRC cell lines could still be dependent on the ERK pathway. This is the case, for instance, in NRAS-mutated melanoma, in which RAF1 and BRAF compensate for each other, and ARAF compensates for the loss of both other isoforms in a mechanism that is still ERK-dependent [6]. Thus, we assessed whether RAF1 ablation might synergize with MEK inhibition in reducing proliferation in 3D cultures, as previously suggested [22]. Treatment of CRC cell lines with the MEK inhibitor U0126 significantly reduced the proliferation of RAF1 proficient spheroids and further reduced spheroid size in RAF1 KO DLD1, SW1116 and KM12 (Fig. 3C). This additive effect was not observed in RAF1-deficient Caco2 clones. Treatment of the parental cell lines with GW5074 in combination with MEK inhibitor did not yield any additive effect, supporting the idea that the unique role of RAF1 in CRC proliferation is kinase-independent (Supplementary Fig. S3C) and implying that in these cells reducing RAF1 expression would be a better therapeutic strategy than targeting its kinase activity.

### Proliferation of CRC patient-derived organoids is RAF1-dependent

**Fig. 4:**
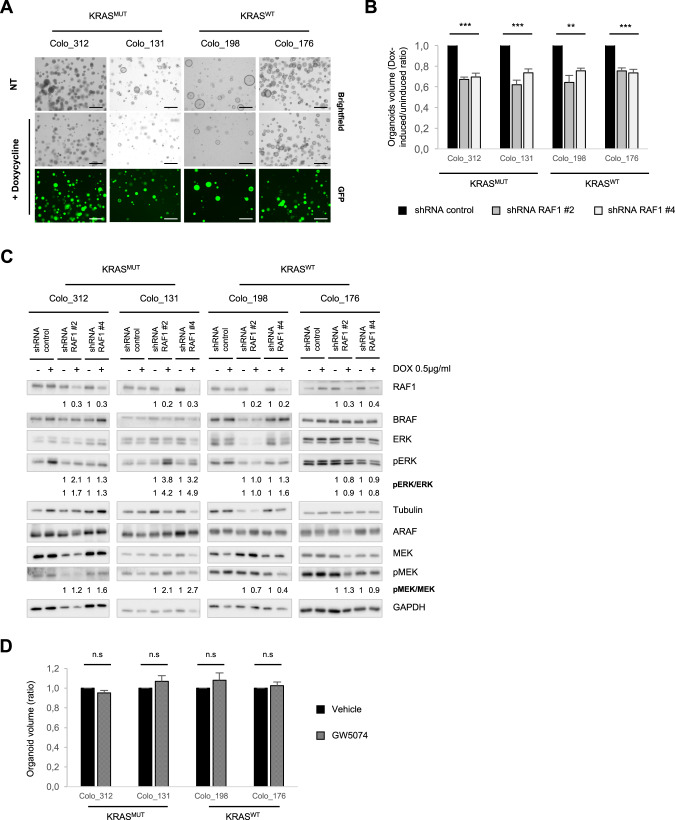
RAF1 depletion impacts patient-derived organoids proliferation. **A** Representative pictures of four patient-derived organoids stably transduced with inducible RAF1 shRNA. Addition of doxycycline (0.5 µg/ml), induces the expression of GFP and of RAF1 shRNA. Scale bars = 250 µm. **B** Comparison of PDOs volume in Dox-induced/uninduced organoids expressing a control shRNA or two independent RAF1 shRNA. Experiments were performed in triplicate at least three times. **C** Immunoblot showing RAF, MEK and ERK status in Dox-induced PDOs. Tubulin or GAPDH were used as loading controls. **D** Patient-derived organoids were treated for 8 days with either DMSO (vehicle) or 1 µM GW5074 prior to end volume determination. The experiment was repeated three times. **B**, **D**, *p < 0.05, **p < 0.01, ***p < 0.001, n.s not significant.

To determine whether our results hold true in patient-derived material, we downregulate RAF1 in patient-derived CRC organoids (PDOs) either by stable expression of two independent doxycycline-inducible RAF1 shRNA (Colo_312 (KRAS^MUT^/MSI; CMS1), Colo_131 (KRAS^MUT^/MSS; CMS not determined), Colo_198 (KRAS^WT^/MSI; CMS4), Colo_176 (KRAS^WT^/MSS; CMS2) or by siRNA (Colo_324 (KRAS^WT^/MSI; CMS3) (Supplementary Table 1). In all cases, silencing of RAF1 significantly reduced the organoid size, independently of the mutational status of KRAS, the MS phenotype, or the CMS classification [31] (Fig. 4A, B and Supplementary Fig. S4A and Supplementary Table 2). ShRNA-mediated RAF1 knock-down (KD) was monitored by immunoblotting (Fig. 4C) and correlated with the expression of GFP, which was induced together with shRNA expression upon doxycycline addition (Fig. 4A lower panel). Overall, MEK and ERK phosphorylation was not decreased but if anything increased upon shRNA-mediated RAF1 depletion in KRAS-mutant PDOs (Fig. 4C), although we observed a slight decrease in MEK phosphorylation in Colo_198 (KRAS^WT^/MSI) and a slight decrease in ERK activity in Colo_176 (KRAS^WT^/MSS) (Fig. 4C). Also similar to the situation in spheroids derived from cell lines, treatment of the PDOs with RAF1 inhibitor GW5074 had no impact on organoid size (Fig. 4D) and had not further effect when used in combination with the MEK inhibitor U0126 (Supplementary Fig. S4B). These results are consistent with a role of RAF1 in CRC proliferation independent of its catalytic activity.

### A RAF1-specific signature reveals new target genes downregulated in human primary tumors

**Fig. 5:**
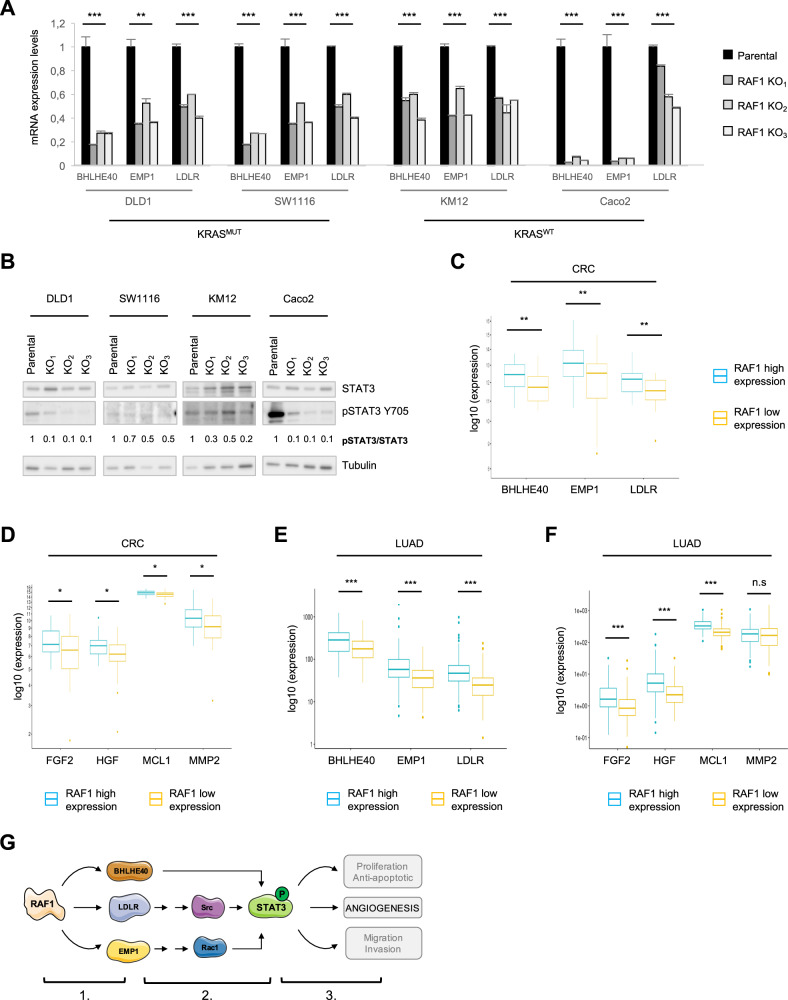
Low expression of RAF1 correlates with the downregulation of target genes involved in STAT3 activation and angiogenesis. **A** mRNA expression levels of *BHLHE40*, *EMP1* and *LDLR* in human CRC spheroids RAF1 WT or KO. **B** Representative STAT3/pSTAT3 western blot of 3D spheroids derived from human CRC cell lines. Numbers represent the ratio between the total STAT3 and the phosphorylated protein, after individual normalization on Tubulin, used as a loading control, and compared to the parental cell line. **C** *BHLHE40*, *EMP1* and *LDLR* expression is significantly downregulated in human primary CRC expressing low levels of RAF1. Comparison was done between RAF1 high expression group (34 patients) and RAF1 low expression group (34 patients). **D** *FGF2*, *HGF*, *MCL1* and *MMP2* expression is significantly downregulated in CRC expressing low levels of RAF1. Comparison was performed as in (**C**). **E** *BHLHE40*, *EMP1* and *LDLR* expression is significantly downregulated in human primary LUAD expressing low levels of RAF1. Comparison was done between the RAF1 high expression group (150 patients) and the RAF1 low expression group (150 patients). **F** Expression of *FGF2*, *HGF* and *MCL1* is significantly downregulated in LUAD expressing low levels of RAF1. Comparison was performed as in (**E**). **G** Working model of RAF1 impact on CRC through BHLHE40, LDLR and EMP1. 1. RAF1 impacts expression of BHLHE40, LDLR and EMP1 via undescribed but ERK-independent mechanisms. 2. BHLHE40, LDLR and EMP1 trigger STAT3 phosphorylation and activation through distinct pathways. 3. Activated STAT3 induces angiogenesis. **A**, **C**, **D**, *p < 0.05, **p < 0.01, ***p < 0.001.

To gain insight in the role of RAF1 in CRC, we performed transcriptomic analysis of 3D spheroids from parental DLD1, SW1116, KM12 and Caco2 cell lines and the three independent RAF1 KO clones derived from them. We selected significantly deregulated genes from each cell line by comparing the three independent clones to their parental cell line (Supplementary Fig. S5A) and identified 12 upregulated and 13 downregulated genes that were common to all four RAF1-deficient CRC cell lines, defining a RAF1-specific signature (Supplementary Fig. S5B). Among the downregulated genes were DUSP6 (Dual Specificity Phosphatase 6), a key negative regulator of ERK [1], and ERRFI1 (ERBB Receptor Feedback Inhibitor 1, also called Mig-6), a tumor suppressor that curbs activation of EGFR and sustained signaling through the ERK pathway [32, 33]. Downregulation of DUSP6 and ERRFI1 might underlie the upregulation of ERK phosphorylation observed in RAF1-deficient spheroids (Fig. 1E and Supplementary Fig. S5B). In addition, three genes implicated in STAT3 activation through distinct pathways [34–36], BHLHE40, LDLR and EMP1, were expressed at low levels in RAF1-deficient CRC spheroids (Fig. 5A). STAT3 is known to be hyperactive in several cancers including CRC, and to control proliferation, metastasis and angiogenesis [37]. Low expression of BHLHE40, LDLR and EMP1 is associated with good prognosis in several cancers, such as pancreatic, urothelial, renal and ovarian cancers [38], while an overexpression of LDLR (Low Density Lipoprotein Receptor) and BHLHE40 (Basic Loop Helix family member e40) is described in colorectal cancer and correlates with poor overall survival [34, 39].

To gain more insight in the pathway(s) impacted by RAF1 ablation, we performed a Proteome Profiler™ Array of human phospho-kinases for the parallel determination of the relative levels of protein phosphorylation. Analysis of the four WT and RAF1 KO CRC lines showed that a number of phosphoproteins were impacted by RAF1 ablation (Supplementary Fig. S6A and raw intensity data in Supplementary Table 3); only 12 phosphosites, however, were affected in all of them (Supplementary Fig. S6B, C). Activating phosphorylation sites [40] on STATs and on STAT3 in particular were prominent among these. While this was the clearest indication of a pathway negatively impacted by RAF1 ablation, we also found a decrease in the activating residues of the SRC kinase family members SRC (Y419) and FGR (Y412), in p53 S15 (important for the DNA damage response and phosphorylated by ERK and p38 [41]), as well as in p38 activation sites, and a decrease in PDGFR Y751 (autophosphorylation site [42]). Increases were found in EGFR Y1086, which creates binding sites for Grb2 and might therefore promote ERK activation [43], Lyn Y397 [44] and in the inhibitory site of GSK3 (S9) [45]. ERK phosphorylation was slightly increased in most of the cell lines, in line with the experiments described in Fig. 1E.

Together, the results of the transcriptomic analysis and of the phosphoproteomic arrays suggested an involvement of STAT3 in the phenotype induced by RAF1 ablation. In line with this, STAT3 phosphorylation on Tyr705 was significantly reduced in RAF1-deficient spheroids (Fig. 5B). Importantly, treatment of RAF1-proficient or -deficient CRC cell lines with the STAT3 inhibitor napabucasin (BBI608) [46] showed that STAT3 inhibition decreased proliferation to levels similar to RAF1 ablation in DLD1 and KM12, while it was more efficient than loss of RAF1 in SW1116 and less efficient in Caco2 cell lines (Supplementary Fig. S7A). However, we did not observe an additive effect of RAF1 loss combined with STAT3 inhibition (Supplementary Fig. S7A). PDOs were very sensitive to napabucasin, which had to be applied in a very low concentration; similar to the situation observed in the spheroids, we could not detect any additive effects of RAF1 depletion and STAT3 inhibition (Supplementary Fig. S7B).

We next tested whether the genes impacted by RAF1 ablation could be correlated with RAF1 expression in an in-house cohort of 135 CRC patients. All patients (stage II and III) had a localized primary tumor resected by surgery within 1 or 2 months after diagnosis and received (stage III) or not (stage II) adjuvant chemotherapy. Patients were divided into four groups (low, mid-low, mid-high and high) according to RAF1 mRNA expression levels (Supplementary Fig. S8A). RAF1 expression levels did not correlate with MSI/MSS phenotype or KRAS mutational status within the MSI group (Supplementary Fig. S8B, C), in agreement with the observations in human CRC spheroids and patient-derived organoids. A trend suggesting that high RAF1 expression is associated with tumor progression within the MSI group was observed (Supplementary Fig. S8D), although it did not reach significance with a Fisher test (*p* = 0.1), probably due to the small number of patients for which we obtained clinical history (Supplementary Table 4). We next selected the low (34 CRC patients) and high (34 CRC patients) RAF1 quartiles and compared the expression of each of the 25 genes found down- or upregulated in the RAF1-deficient CRC spheroids. Of these, only BHLHE40, LDLR and EMP1 were significantly downregulated in CRC tumors expressing low levels of RAF1 (Fig. 5C). We therefore investigated the expression of STAT3 targets in our patient cohort according to the distribution of RAF1. Remarkably, four STAT3 target genes that enhance angiogenesis (*FGF2*, *HGF*, *MCL1* and *MMP2*) [47–52] were significantly downregulated in tumors expressing low levels of RAF1 (Fig. 5D). To determine whether the RAF1-STAT3 axis is restricted to CRC, we correlated the expression of RAF1 with that of these four genes in lung adenocarcinoma (LUAD), in which RAF1 is known to have kinase-independent functions [11, 17]. Analysis of the 600 cases from the TCGA LUAD cohort subdivided in quartiles according to RAF1 expression (Supplementary Fig. 8E) revealed a significant downregulation of *BHLHE40*, *EMP1* and *LDLR* as well as of three STAT3 target genes (*FGF2*, *HGF* and *MCL1*) in tumors expressing low levels of RAF1 (Fig. 5E, F). These observations are similar to those made in the CRC cohort (Fig. 5C, D). Taken together, these results suggest that the RAF1-STAT3 axis could be a general signaling pathway in cancer cells (Fig. 5G).

## Discussion

In the present study, we investigated the role of RAF1 in colorectal cancer by using human 3D models as spheroids from CRC cell lines and patient-derived organoids. First, we demonstrated that complete ablation of RAF1 by CRISPR/cas9 genome engineering or RAF1 silencing with shRNA reduces the proliferation of human colorectal cancer cell lines and patient-derived organoids, respectively. This is in line with previous studies reporting a role of RAF1 in CRC [22, 23], lung adenocarcinoma [9, 10], pancreatic cancer [7, 53] and squamous cell carcinoma [8]. CRC studies widely focus on KRAS mutated CRC and MSS CRC [22, 23]. By screening 2D and 3D cultures of CRC cell lines and PDOs with different KRAS genotype, MS phenotype and molecular subtypes, we demonstrate that the role of RAF1 is independent of KRAS mutational status, MS phenotype and CMS classification. Over the last years, it has been well established that RAF1 exerts MEK-independent functions, including anti-apoptotic functions [54, 55], regulation of cell cycle and mitosis checkpoints [14, 15] and a role in cell migration [16]. In CRC cell lines, as previously described, RAF1 ablation does not affect apoptosis [23] but rather cell cycle, independently of KRAS genotype.

In CRC colonospheres [23], inhibition of RAF1 kinase activity with the specific inhibitor GW5074 reduced clone-forming capacity and restored the polarity. The same inhibitor in our study did not reveal an effect on the proliferation of CRC spheroids or patient-derived organoids, indicating that kinase activity of RAF1 is not necessary for cell proliferation. In line with this, RAF1 WT and a kinase-dead RAF1 mutant rescued the proliferation phenotype to similar extents. Thus, in agreement with results obtained in lung adenocarcinomas also with RAF1 kinase-dead reconstitution experiments [11, 17], RAF1 kinase activity is dispensable for cell proliferation in CRC spheroids and organoids. Further mutational analysis revealed that the phosphoablative RAF1 S338A, which prevents growth factor-mediated phosphorylation and activation, rescued the defect in proliferation, while the dimer interface mutant DIF, which prevents RAF dimerization, failed to do so. Dimerization has been shown to promote RAF1 S338 phosphorylation; [56] in turn, S338 phosphorylation is necessary for full-fledged activity in the context of RAF dimers [57]. This is possibly due to the fact that in the context of the dimers, only phosphorylated RAF1 molecules can allosterically activate the other component, while phosphoablative mutants can only function as receivers [58]. Together with the normal levels of ERK phosphorylation observed in all RAF1 KO CRC cell lines/PDO and with the rescue of the RAF1 phenotype by a kinase-dead mutant (functional as an activator) the data indicate that the role of RAF1 in CRC is independent of its kinase activity, and that the role of the RAF1 dimerization interface is unlikely to be rooted in its effect on the ERK pathway. Instead, stoichiometry appears to be crucial, as shown by the effect of even partial reduction of RAF1 expression on the proliferation of CRC spheroids and organoids. The results imply that targeting RAF1 using selective degraders could be beneficial in CRC independently of their KRAS genotype and microsatellite phenotype.

In addition and in line with similar studies performed with RAF1 shRNA in pancreatic cancer [7], bladder tumors [59], lung adenocarcinoma [17] and CRC [22], we demonstrated that a combination of RAF1 ablation and MEK inhibition greatly impairs cell proliferation in 3D spheroids from human CRC cell lines and CRC patient-derived organoids, confirming that combination therapies might be a strategy in CRC. A further possibility for combination therapies is suggested by the combined results of the phosphosite profiler arrays and of the transcriptomic analysis carried out in RAF1-proficient and -deficient CRC spheroids. Phosphosite analysis indicated an impact of RAF1 on a rather limited number of pathways, including two RTKs (PDGFRβ and EGFR, inversely affected), a rewiring of SRC family kinases, and decreased activity of the p38–p53 axis. STAT activation was the pathway most clearly affected by RAF1 loss. In the transcriptomic analysis, we discovered that RAF1 ablation downregulates *BHLHE40, LDLR or EMP1*. Importantly, we could establish a positive correlation between the expression levels of these genes and those of RAF1 expression in human primary CRC. *BHLHE40, LDLR and EMP1* encode proteins that enhance STAT3 activation [34–36]. Analysis of STAT3 activation in RAF1-deficient CRC spheroids and of the expression of STAT3 target genes in CRC patients confirmed a positive correlation between RAF1 expression and STAT3 activity. In particular, RAF1 expression correlated with the expression of STAT3 targets involved in the control of angiogenesis [47–52], a crucial process necessary for tumor growth and metastasis development. Similarly, we established a positive correlation between RAF1 expression and *BHLHE40, LDLR and EMP1*, as well as the STAT3 target genes involved in the control of angiogenesis in human primary LUAD. RAF1 exerts dual roles in STAT3 activation; in hepatocellular carcinoma, RAF1 acts as a cell-autonomous tumor suppressor by negatively regulating STAT3 activation [60], while it has been described as a STAT3 activator in squamous cell carcinoma through its interaction with ROKα [8]. Here, we demonstrate a positive correlation between RAF1 expression and STAT3 activation in CRC and LUAD, suggesting that RAF1 interaction partners and functions are cell-type and tissue specific [61, 62].

Be that as it may, low expression of BHLHE40, LDLR or EMP1 and a weak STAT3 activity are associated with a good prognosis in several cancers [37, 38], and STAT3 inhibitors such as Napabucasin (BBI608) are FDA-approved in pancreatic cancer and give promising results in CRC clinical trials, alone or in combination [46, 63]. The effect of STAT3 inhibition is more or less comparable to that of RAF1 loss. However, we did not observe any additive effects of RAF1 loss combined with STAT3 inhibition in CRC cell lines or PDOs. Based on our data, one could hypothesize that co-targeting RAF1 expression with selective degraders and STAT3 itself is not a promising strategy, while co-targeting RAF1 expression and MEK/ERK activation might show therapeutic efficacy.

## Material and methods

### Cell culture

Colon cancer cell lines were cultured in DMEM high glucose (4.5 g/l, Gibco, Thermo Scientific Inc., Waltham, MA) supplemented with 10 % FBS, 100 U/ml penicillin G, and 100 lg/ml streptomycin at 37 °C and 5% CO_2_.

Spheroid cultures were established as described [26] with 3000 cells per well. Images were acquired with an inverse microscope (Olympus IX83) equipped with a camera (Hamamatsu Flash4, Hamamatsu, Japan) and analyzed with the ImageJ software (NIH, National Institute of Health, Bethesda, Maryland, USA). Spheroid volume was determined by measuring projected areas, followed by radius and volume (μm³) determination.

Patient-derived organoids were cultured as previously described [64] in 80% Cultrex RGF BME Type 2 (R&D Systems #3533-005).

### CRISPR/cas9 screening

Generation of RAF1 KO cell lines was performed by the ProTech facility at Vienna BioCampus Facility. Briefly, CRC cell lines were electroporated with cas9 protein and pSpCas9(BB)-2A-GFP (PX458) plasmid (gift from Feng Zhang, MIT, Cambridge; Addgene plasmid # 48138; RRID:Addgene_48138)) [65] containing two different RAF1 guide RNAs (♯sgRAF1_1: TGCATCAATGGAGCACATAC; ♯sgRAF1_2: GCTTGGAAGACGATCAGCAA). Single cell colonies were grown in complete medium and selected for RAF1 depletion by Western Blot.

### Crystal violet staining

Cells were plated in 96-well plates and were fixed for 15 min with ice-cold methanol at day 5, washed with PBS and stained with 0.5% Crystal Violet for 30 min at RT. After three washes with PBS, plates were air-dried and Crystal Violet was eluted with 10% Acetic Acid. Absorbance was measured at 595 nm using a microplate reader (Tecan^®^, Austria).

### Colony-forming assay

Cells were plated at low density (1000 cells/well) in 6-well plates and let grow for 12 days. After Crystal Violet staining (see above), plates were air-dried and colonies were counted.

### Immunoblot analysis

Whole cell lysates from spheroids or organoids were extracted in RIPA lysis buffer (50 mM Tris pH 7.6, 150 mM NaCl, 1% Triton X-100, 0.1% SDS, 0.5% sodium-deoxycholate) supplemented with PMSF 1 mM, NaF 1 mM, Na3VO4 1 mM and protease cocktail inhibitor 1X (Roche, #P8849). For SDS-Page analysis, PVDF membranes were incubated overnight at 4 °C with the appropriated primary antibodies, all diluted 1:1000: BRAF F-7 (#sc-5284), ARAF C-20 (#sc-408), β-actin (#sc-1616), all from Santa Cruz Biotechnology, RAF1 (#610152) from BD Biosciences, MEK (#9122), pMEK (#9121), ERK (#9102), pERK (#9101), pSTAT3 Tyr705 (#9145), STAT3 (#8768) and GAPDH (#5174) from Cell Signaling Technology and Tubulin (#T9026) from Sigma-Aldrich. Immunoblots were acquired using ChemiDoc Imaging Systems and quantified using the Image Lab software (Bio-Rad Laboratories, Hercules, USA).

### Inhibitor treatment

Spheroids and patient-derived organoids were treated with 1 µM or 10 µM U0126 (Cell signaling #9903; concentrations stated in the figure legend) and/or 1 µM GW5074 (MedChemExpress #HY-10542), or with napabucasin (BBI608) (MedChemExpress #HY-13919) at the indicated concentrations. Pictures were taken at the beginning and at the end of treatment to calculate the ratio between the initial and the endpoint volume.

### Lentiviral transduction

For silencing experiments, two different shRNA sequences were cloned in the LT3GEPIR vector (gift of Johannes Zuber, IMP, Vienna; Addgene plasmid # 111177; RRID: Addgene_111177) [66].

shRNA-2: TGCTGTTGACAGTGAGCGCTGGCACGGAGATGTTGCAGTATAGTGAAGCCACAGATGTATACTGCAACATCTCCGTGCCATTGCCTACTGCCTCGGA

shRNA-4: TGCTGTTGACAGTGAGCGATCCCTCAATTATGTTATTTTATAGTGAAGCCACAGATGTATAAAATAACATAATTGAGGGACTGCCTACTGCCTCGGA

For reconstitution experiments, RAF1 full length (RAF1 WT), kinase-dead (D486A), phosphoablative (S338A) or dimer deficient (DIF) mutants [30] were cloned in pLJM1-EGF vector (gift of David Sabatini, Whitehead Institute, Cambridge; Addgene plasmid # 19319; RRID: Addgene_19319) [67]. Lentiviruses were produced in HEK293T cells by co-transfecting the vectors with VSV-G and gag-pol plasmids using PEI. Lentiviral particles were harvested 48 h post-transfection and directly added to the cells.

### siRNA transfection

Lipofectamine RNAiMax (Invitrogen) was used to transfect the CRC cell line LS174T and the PDO Colo_324 with 30 nM RAF1 siRNA (Sigma-Aldrich, NM_002880 (ID: SASI_Hs01_00174876) [60, 68]. esiRNA against Renilla Luciferase (Sigma-Aldrich, RLuc, EHURLUC) was used as negative control.

### Cell cycle, apoptosis and metabolic activity assays

Cell cycle analysis was carried out using the Click-iT^®^ EdU Alexa Fluor 488 Kit (Invitrogen, Thermo Scientific Inc., Waltham, MA), following the manufacturer’s instructions. Spheroids were exposed to EdU (5-ethynyl-2’-deoxyuridine, 10 μM) at 37 °C for 2 h. Thereafter, spheroids were trypsinized and the cells were FACS-analyzed. Dapi was used to stain total DNA and forward scatter to determine cell size.

Apoptosis was assessed by flow cytometry using the Annexin A5/7-AAD kit (Beckman Coulter, #IM3614). The metabolic capacity of cells was determined using CellTiter-Glo^®^ 3D (Promega, Madison, WI, #G9682) according to the manufacturer’s protocol. Luminescence was measured with a microplate reader (Tecan^®^, Austria).

### Quantitative PCR

RNA was extracted from 3D spheroids with the RNeasy kit from Qiagen (#74104) following manufacturer’s instructions, and reverse transcribed with the High-Capacity cDNA Reverse Transcription Kit (Applied Biosystems, #4368813). qPCR was performed in the Applied Biosystems 7900HT Fast Real-Time PCR System, using Go Taq qPCR Master mix (Promega, #A6001). Relative expression was calculated by the ΔΔCT method using *ACTIN* as housekeeping gene. The primers are from Eurofins (Nantes, France) (Supplementary Table 5).

### Immunohistochemistry

For histological analysis, spheroids were fixed in 4% paraformaldehyde, molded in 1% agarose gel and embedded in paraffin. Hematoxylin/eosin and KI67 staining were performed on 4 µm-thick paraffin sections. Antigen retrieval in citrate buffer (pH6.0) was performed before immunostaining, and sections were treated with 3% H_2_O_2_-PBS to inhibit endogenous peroxidase. After blocking in 3% BSA-PBS, sections were incubated for 1 h at room temperature with antibodies against Ki67 (Thermo Scientific, #RM-9106-S) or cleaved caspase-3 (Cell signaling Technology, #9661), washed, incubated with the Leica Bond Polymer Detection kit (Leica, #DS9800) and counterstained with hematoxylin.

### Proteome Profiler™ array

Levels of phosphorylated sites were determined using the Proteome Profiler™ Array, Human Phospho-Kinase Array Kit (R&D Systems # ARY003C). In total, 100 µg of protein lysates were incubated on the membranes spotted with antibodies specific of 37 kinase phosphorylation sites. Experiment was performed according to manufacturer’s recommendations. After background substraction, average of the duplicated spots was normalized on the loading control (raw intensity data are available in Supplementary Table 5) and the log2 ratio to the parental cell line was calculated.

### RNA sequencing and analysis (human CRC cell lines)

mRNA library preparation was realized following manufacturer’s recommendations (Kapa mRNA Hyperprep, Roche). Final samples pooled library prep was sequenced on ILLUMINA Novaseq6000 with SP-100 cartridge (2 × 800 Millions of 50 bases reads), corresponding to 2 × 33 Millions of reads per sample after demultiplexing.

The quality of raw data was evaluated with FastQC (http://www.bioinformatics.babraham.ac.uk/projects/fastqc). Poor quality sequences were trimmed or removed with fastp software to retain only good quality paired reads. Star v2.5.3a [69] was used to align reads on hg19 reference genome using standard options. Quantification of gene and isoform abundances has been done with rsem 1.2.28 [70]. Data were normalized with edgeR (v3.28.0) bioconductor packages [71], prior to differential analysis with glm framework likelihood ratio test from edgeR package workflow.

### Human primary CRC samples (in-house cohort)

This study included 135 MSI/MSS cases from a prospective series of patients who underwent surgical resection of mainly stage II-III CRC from 2004 to 2015 at Saint-Antoine Hospital, Paris, France. MSI status was identified prospectively at diagnosis using the pentaplex PCR method. Patients who received preoperative chemotherapy and/or radiotherapy were excluded. Some stage II patients and most of the stage III patients received adjuvant chemotherapy. OS and RFS were evaluated 5-year after diagnosis or surgical resection and only for MSI patients. This study was approved by the institutional review boards/ethic committee of Saint-Antoine Hospital and informed consent was recorded.

### Human primary LUAD samples

This study included analysis of transcriptomic data from 600 cases of LUAD obtained from TCGA repository. https://portal.gdc.cancer.gov/repository?facetTab=files&filters=%7B%22op%22%3A%22and%22%2C%22content%22%3A%5B%7B%22content%22%3A%7B%22field%22%3A%22cases.project.project_id%22%2C%22value%22%3A%5B%22TCGA-LUAD%22%5D%7D%2C%22op%22%3A%22in%22%7D%2C%7B%22op%22%3A%22in%22%2C%22content%22%3A%7B%22field%22%3A%22files.access%22%2C%22value%22%3A%5B%22open%22%5D%7D%7D%2C%7B%22content%22%3A%7B%22field%22%3A%22files.experimental_strategy%22%2C%22value%22%3A%5B%22RNA-Seq%22%5D%7D%2C%22op%22%3A%22in%22%7D%5D%7D&searchTableTab=files.

### RNA sequencing and analysis (in-house cohort)

Frozen tissue sections were lysed in QIAzol Lysis Reagent using a TissueLyzer (Qiagen). After chloroform separation, RNA extractions were performed using the miRNeasy Mini Kit (Qiagen) on a QIACube instrument (Qiagen) following the manufacturer’s instructions including DNAse treatment. RNA integrity was assessed on a Bioanalyzer 2100 (Agilent), the average RIN (RNA Integrity Number) calculated for tumor and normal adjacent tissues was equal 8.1 and 7.2, respectively. Downstream RNA sequencing experiments were performed on samples with a RIN ≥ 7. mRNA sequencing has been performed by the «Centre National de Recherche en Génomique Humaine, Institut de Biologie François Jacob, CEA». Libraries were prepared using the “TruSeq Stranded mRNA Library Prep Kit” from Illumina, according to the manufacturer’s instructions and with an input of 1 µg (selection of poly(A) RNAs). Sequencing was performed on an Illumina HiSeq 2000 sequencer as paired-end 100 bp reads. Libraries were generally pooled by 4 samples per lane. Sequencing quality control was checked with FASTQC and reads were then aligned against GRCh38 reference genome using Star v2.7.2.

Gene counts were obtained using htseq-count, normalized by an UpperQuartile procedure and logged on a base 2.

### Statistical analysis

Quantitative data are presented as mean ± SEM of at least three independent experiments, as indicated in the figure legends. *p* values were calculated using the Student’s *t* test (two-tailed), and were considered statistically significant when <0.05. For transcriptomic analysis on human CRC spheroids, multiple hypothesis adjusted *p* values were calculated with the Benjamini–Hochberg procedure to control FDR which was set up with a significance of 0.05. For proteome profiler™ array analysis, a two-tailed Student’s *t* tests were performed between the log2 ratios of the parental cell lines and log2 ratios of RAF1 KO cell lines for each antibody. *p* values were adjusted using the Benjamini–Hochberg method. The heatmap was plotted with the pheatmap package v1.12.0 (https://CRAN.R-project.org/package=pheatmap) using R.

Statistical analysis of the gene expression experiments (in-house cohort and LUAD TCGA cohort) was performed using R and gene expression comparisons were performed using the Wilcoxon Mann–Whitney test with a significance cut-off of 0.05.

## Supplementary information

Supplemental information

Supplementary Table 3

## Data Availability

All datasets generated during and/or analyzed during the current study are available from the corresponding author on reasonable request.
