# Supplementary material for: RAF1 contributes to cell proliferation and STAT3 activation in colorectal cancer independently of microsatellite and KRAS status
Source: Oncogene. 2023 Apr 5;42(20):1649–60. doi: 10.1038/s41388-023-02683-w (PMC10181936; doi:10.1038/s41388-023-02683-w)
Supplement: Supplementary file 1 — Supplemental information [file 41388_2023_2683_MOESM1_ESM.pdf]

Cell lines	CMS consensus classification (24, 25)	CMS frequency (25)	MS phenotype	KRAS status	BRAF status	PTEN status	PIK3CA status	APC status	TP53 status
DLD1	CMS4	14%	MSI	G13D	WT	positive	MUT	MUT	MUT
SW1116	CMS2	37%	MSS	G12A	WT	positive	WT	WT	MUT
KM12	CMS3	14%	MSI	WT	WT	null	WT	MUT	MUT
Caco2	CMS4	23%	MSS	WT	WT	positive	WT	MUT	MUT
LS174T	CMS1	13%	MSI	G12D	WT	positive	MUT	WT	WT
**Patient-derived organoids**	**CMS consensus classification prediction (31)**	**CMS frequency (25)**	**MS phenotype**	**KRAS status**	**BRAF status**	**PTEN status**	**PIK3CA status**	**APC status**	**TP53 status**
Colo_312	CMS1	14%	MSI	G13A	WT	n.d	MUT	WT	WT
Colo_131	n.d		MSS	G12V	WT	n.d	MUT	MUT	WT
Colo_198	CMS4	23%	MSI	WT	WT	n.d	MUT	MUT	MUT
Colo_176	CMS2	37%	MSS	WT	WT	n.d	WT	MUT	MUT
Colo_324	CMS3	13%	MSI	WT	WT	n.d	MUT	MUT	WT

WT: wild-type, MUT: mutated, MSI: microsatellite instable, MSS: microsatellite stable, n.d: not determined

Supplementary Table 1. Characteristics of CRC cell lines and patient-derived organoids.

Supplementary Table 1

Cell lines	CMS consensus classification (24, 25)	CMS frequency (25)	MS phenotype	KRAS status	Effect of RAF1 KO1	Effect of RAF1 KO2	Effect of RAF1 KO3	Effect of RAF1 KD shRNA #2	Effect of RAF1 KD shRNA #4	Effect of RAF1 KD siRNA
DLD1	CMS1	14%	MSI	G13D	0.47 ± 0.03	0.55 ± 0.02	0.43 ± 0.04	0.77 ± 0.02	0.85 ± 0.02	
SW1116	CMS2	37%	MSS	G12A	0.67 ± 0.06	0.66 ± 0.05	0.6 ± 0.02			
KM12	CMS1	14%	MSI	WT	0.42 ± 0.03	0.34 ± 0.01	0.3 ± 0.04			
Caco2	CMS4	23%	MSS	WT	0.51 ± 0.07	0.66 ± 0.04	0.6 ± 0.05			
LS174T	CMS3	13%	MSI	G12D				0.81 ± 0.04	0.90 ± 0.02	0.87 ± 0.02
**Patient-derived organoids**	**CMS consensus classification prediction (31)**	**CMS frequency (25)**	**MS phenotype**	**KRAS status**						
Colo_312	CMS1	14%	MSI	G13A				0.67 ± 0.02	0.7 ± 0.04	
Colo_131	n.d		MSS	G12V				0.62 ± 0.04	0.74 ± 0.04	
Colo_198	CMS4	23%	MSI	WT				0.64 ± 0.07	0.76 ± 0.03	
Colo_176	CMS2	37%	MSS	WT				0.76 ± 0.03	0.74 ± 0.03	
Colo_324	CMS3	13%	MSI	WT						0.85 ± 0.03

WT: wild-type, MUT: mutated, n.d: not determined, KO: knockout, KD: knockdown
Data are represented in KO/WT ratio or KD/WT ratio ± SEM

Supplementary Table 2. Effect of RAF1 KO and KD on spheroid and patient-derived organoid proliferation.

Supplementary Table 2

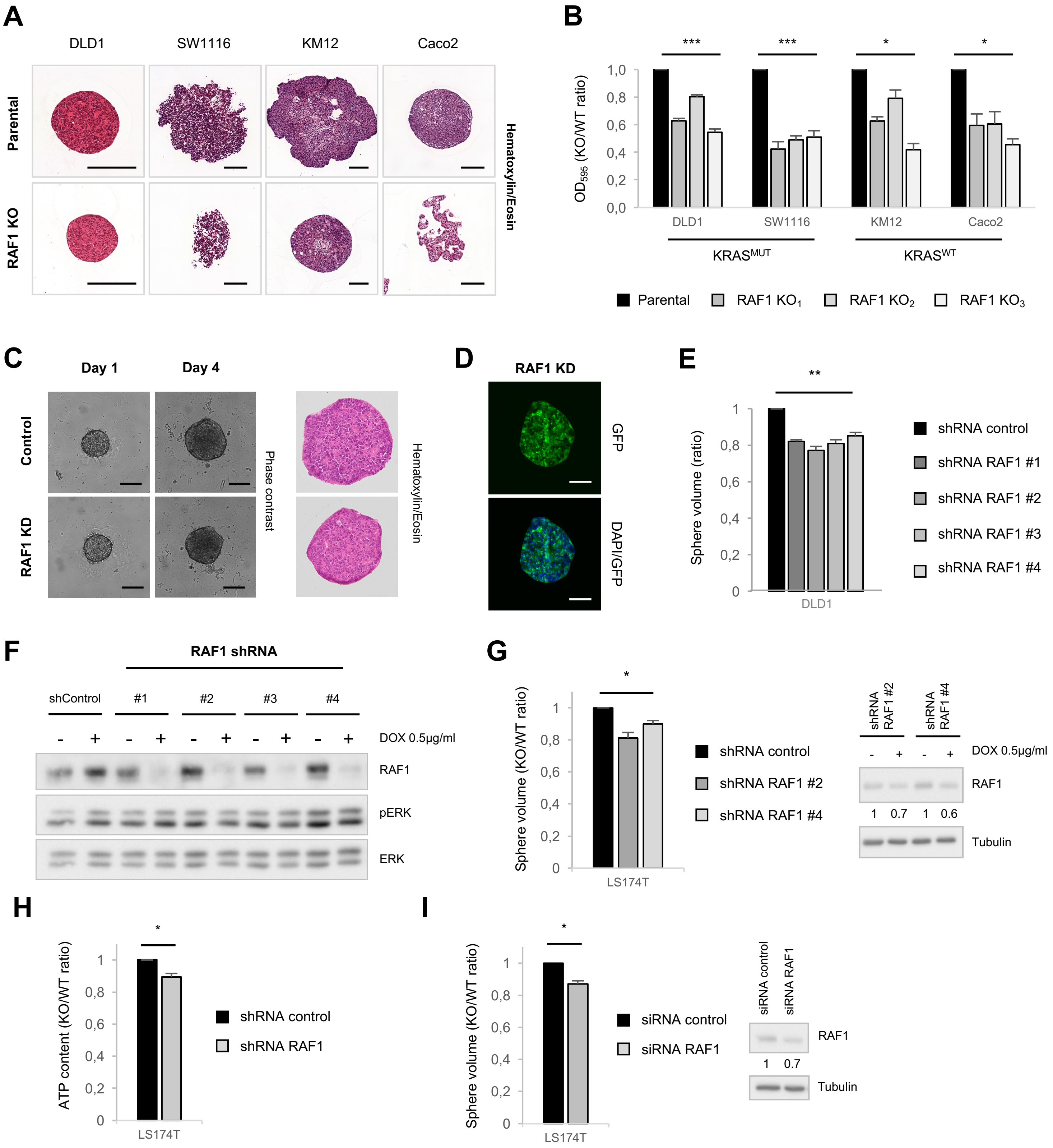

Supplementary Figure 1. RAF1 ablation does not impact spheroid morphology, reduces cell proliferation of 2D cultures and RAF1 silencing slows down cell proliferation in CRC cell lines. **A**, Hematoxylin/Eosin staining of 3D spheroids 5 days after spheroid formation. RAF1 KO pictures are representative of the three independent KO clones. Scale bars = 200µm. **B**, Proliferation in 2D cultures was measured by Crystal Violet staining 5 days after plating (n= 3 separate experiments). **C**, Growth of DLD1 spheroid stably transduced with shRNA control or RAF1 shRNAs in the absence (control) or presence of doxycycline (RAF1 KD). Left panel, phase contrast images; right panel, Hematoxylin/Eosin staining. Scale bars = 250µm **D**, Fluorescent images of spheroids upon doxycycline treatment revealed expression of GFP in the entire spheroid. Expression of GFP is correlated with shRNA expression. Scale bars = 100µm. **E**, DLD1 spheroid volume upon RAF1 KD, obtained with 4 different shRNAs. **F**, Western blot showing silencing efficacy in DLD1 cell line. ERK was used as a loading control. **G**, LS174T spheroid volume upon RAF1 KD, obtained with 2 different shRNAs, 5 days after doxycycline induction. Experiment performed three times (left panel). Western blot showing silencing efficacy in LS174T cell line (right panel). Tubulin was used as a loading control. **H**, ATP content 5 days after spheroid formation of LS174T cell line and RAF1 silencing, representing three independent experiments. **I**, LS174T spheroid volume upon RAF1 KD, obtained with siRNA 5 days after transfection, experiment performed three times (left panel). Immunoblot showing silencing efficacy in LS174T cell line (right panel). Tubulin was used as a loading control. **B, E, G-I**, p*<0.05, p**<0.01, p***<0.001, n.s not significant.

Supplementary Figure 1

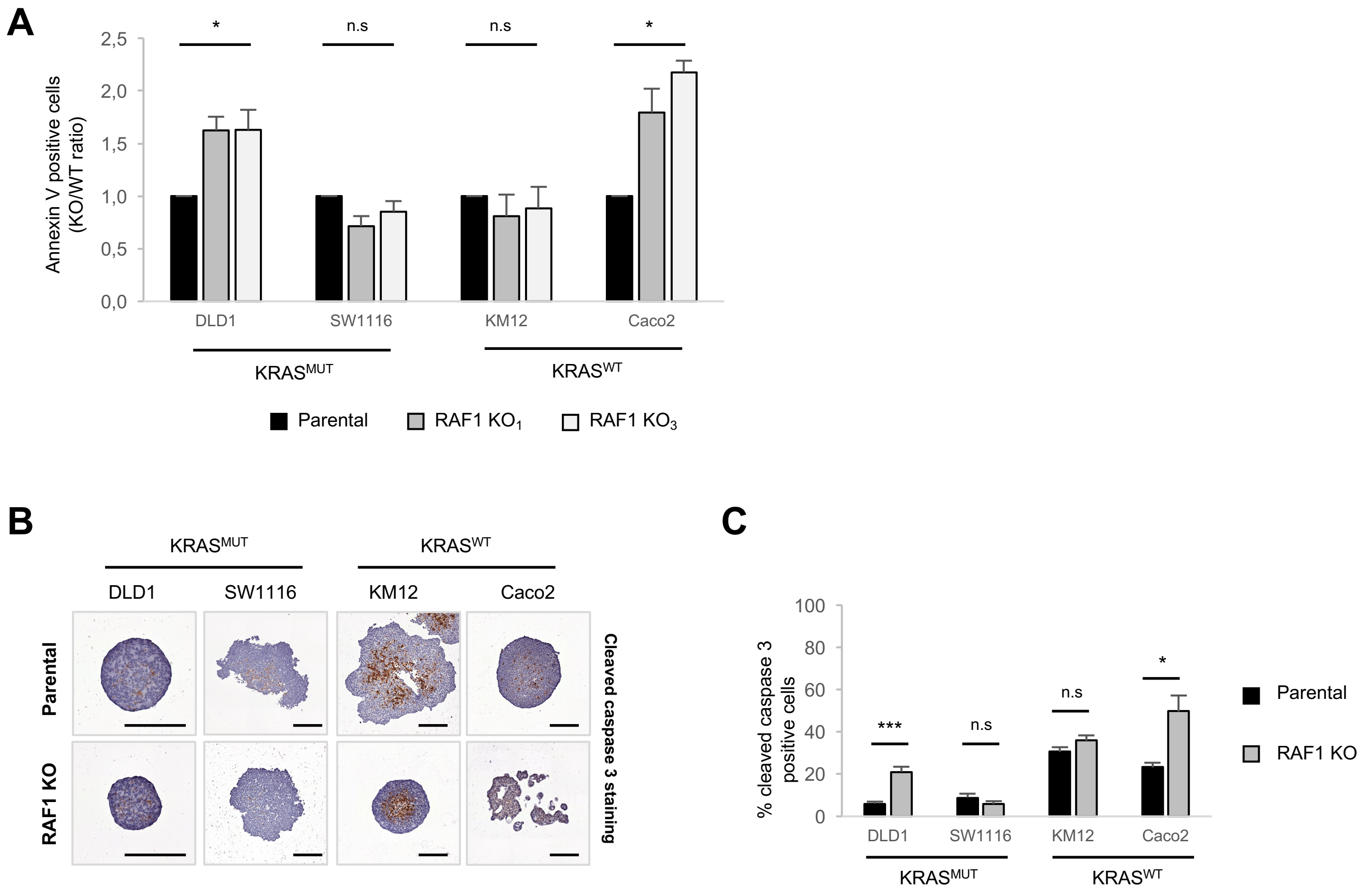

Supplementary Figure 2. RAF1 loss promotes apoptosis. **A**, 5 days after spheroid formation, spheroids were dissociated and the number of apoptotic cells (annexin V-positive cells) was determined by flow cytometry. The experiment was performed at least 3 times on two RAF1 KO independent clones per cell line. **B**, Cleaved-caspase 3 staining of spheroids after 5 days in culture. KO pictures are representative of 3 independent clones/cell line. Scale bars=200µm. **C**, Quantification of cleaved caspase 3-positive cells in parental and RAF1 KO spheroids. **A and C**, p*<0.05, p**<0.01, p***<0.001, n.s not significant.

Supplementary Figure 2

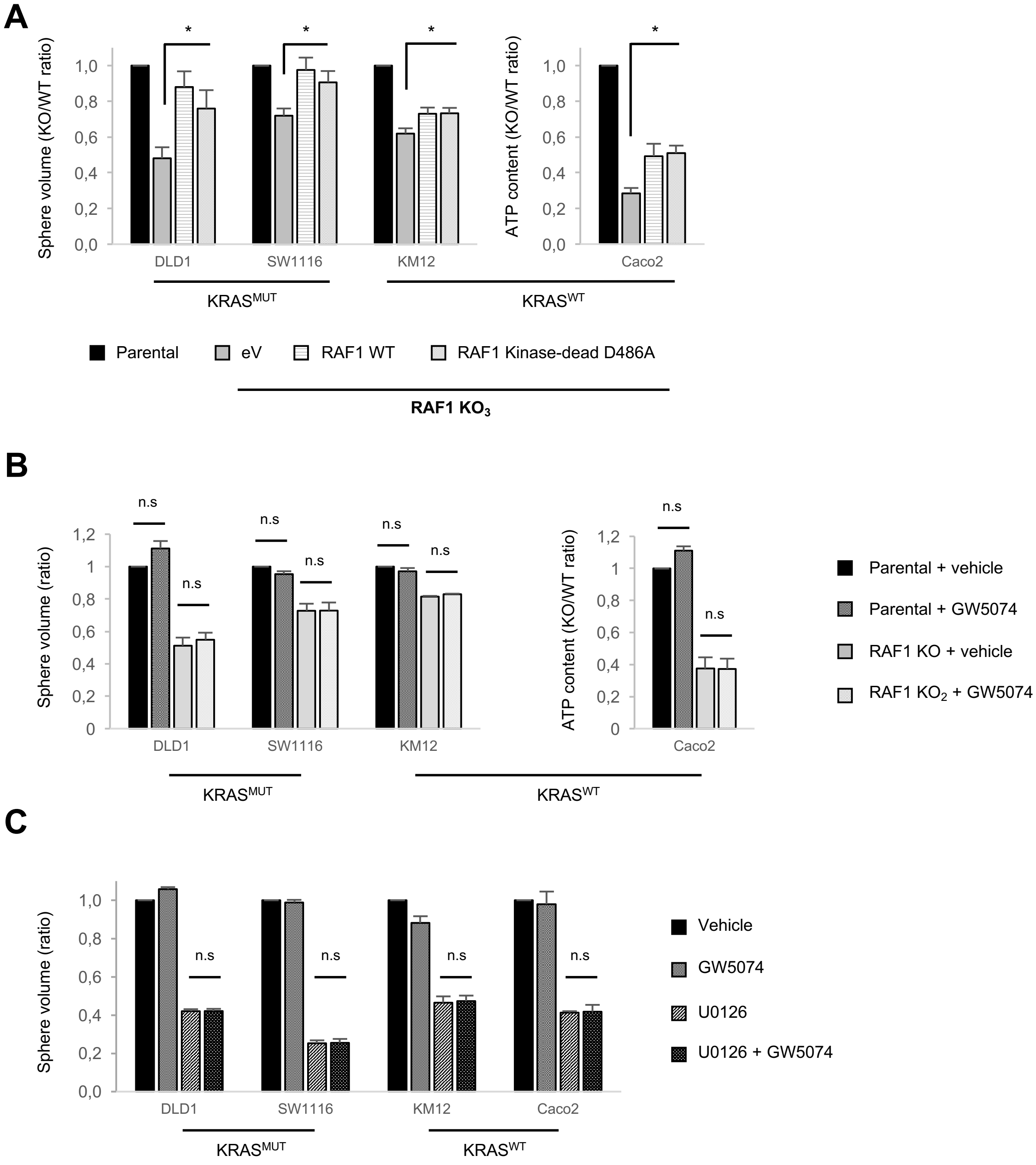

Supplementary Figure 3. RAF1 kinase activity is not required for proliferation and its inhibition is not enhanced by MEK inhibition. **A**, Spheroid volume or ATP content were determined 5 days after spheroid formation by comparing the parental cell line with the RAF1 KO_3 clone reconstituted with empty vector or with the RAF1 constructs. Experiment was performed at least three times. **B**, Spheroids from CRC cell lines parental and RAF1 KO were treated with either DMSO (vehicle) or 1μM GW5074 for 5 days, and volume was measured at the end of the experiments, performed in triplicate. **C**, Spheroids from parental CRC cell lines were treated for 5 days with either DMSO (vehicle), 10μM U0126 (DLD1, SW116 and Caco2) or 1μM U0126 (KM12), alone or in combination with 1μM GW5074. Volume of spheroids was measured at the end of the experiment which was performed in triplicate. **A-C**, p*<0.05, p**<0.01, p***<0.001, n.s not significant.

Supplementary Figure 3

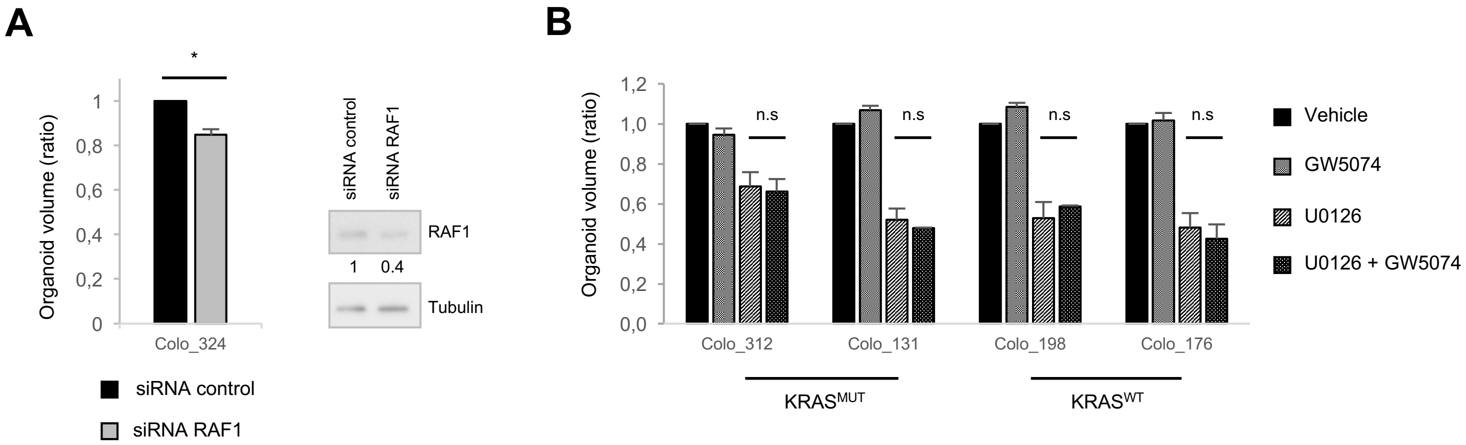

Supplementary Figure 4. RAF1 depletion by siRNA slows down patient-derived organoid proliferation and RAF1 kinase activity is dispensable in PDOs. **A**, Colo_324 organoid volume upon RAF1 KD, obtained with siRNA, experiment performed three times (left panel). Immunoblot showing silencing efficacy in Colo_324 PDO (right panel). Tubulin was used as a loading control. **B**, Patient-derived organoids were treated either with DMSO (vehicle), GW5074 (1µM) or U0126 (10µM for Colo_198 and Colo_176 or 1µM for Colo_312 and Colo_131), alone or in combination. Volume of PDOs was measured after 8 days of treatment. Experiments were performed in triplicate. **A and B**, p*<0.05, p**<0.01, p***<0.001. n.s not significant

Supplementary Figure 4

A

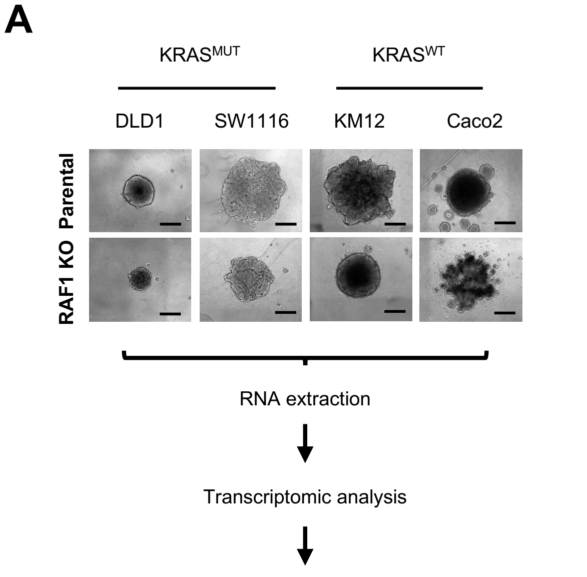

Cell line	Upregulated genes	Downregulated genes
DLD1 RAF1 WT versus RAF1 KO	130	188
SW1116 RAF1 WT versus RAF1 KO	1880	1392
KM12 RAF1 WT versus RAF1 KO	755	556
Caco2 RAF1 WT versus RAF1 KO	3826	3386

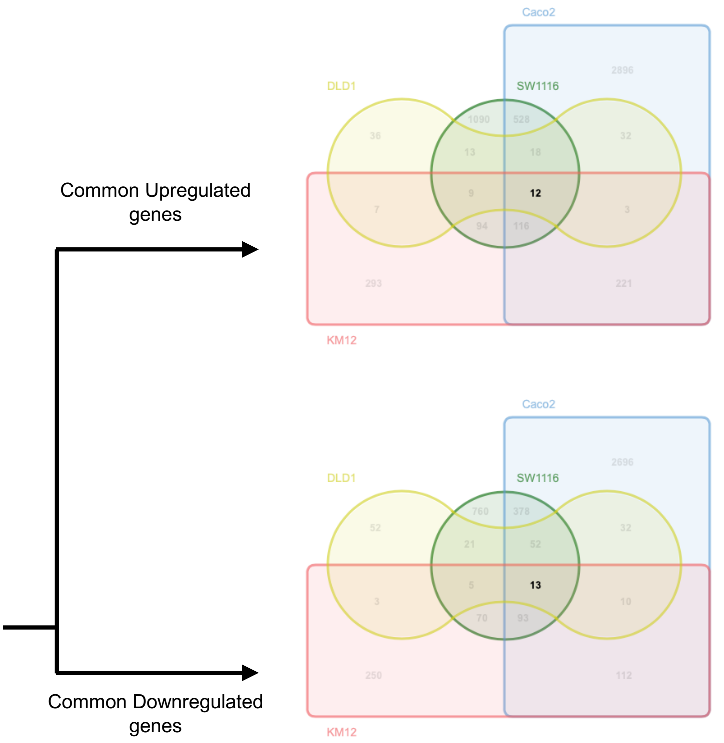

B

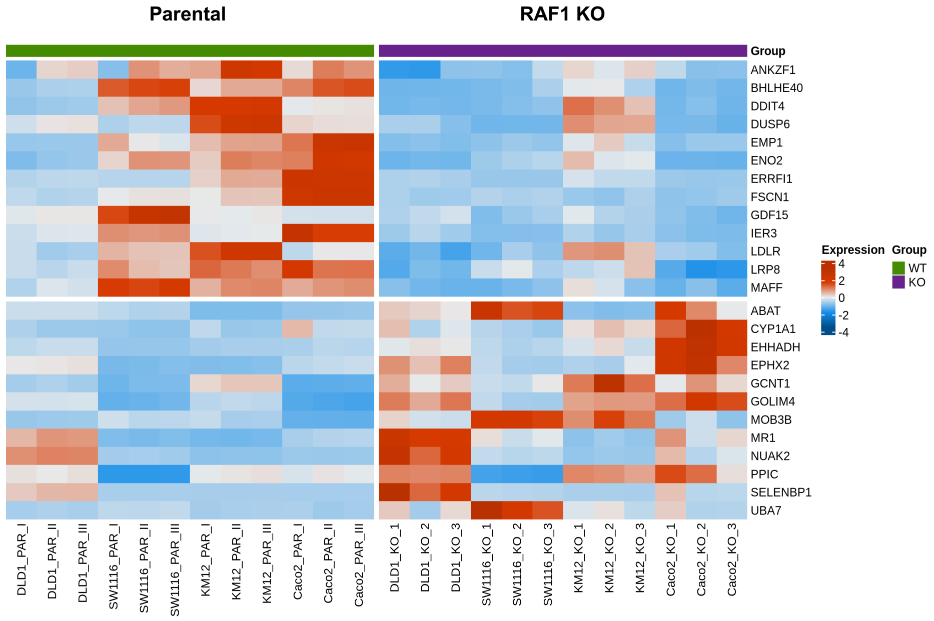

Supplementary Figure 5. RAF1 transcriptomic signature in human CRC cell lines. **A**, Schematic representation of the experimental procedure. 3D spheroids from cell lines were collected and mRNA was extracted 5 days after spheroid formation. Transcriptomic analysis revealed upregulated and downregulated genes in absence of RAF1 for each cell line. Venn diagram identified upregulated and downregulated genes common to all four cell lines (right panel). **B**, Heatmap of the deregulated genes comparing parental cell lines (left panel) with RAF1 KO cell lines (right panel) with a 0.5 log2 fold-change and a FDR-adjusted p-value at 0.05.

Supplementary Figure 5

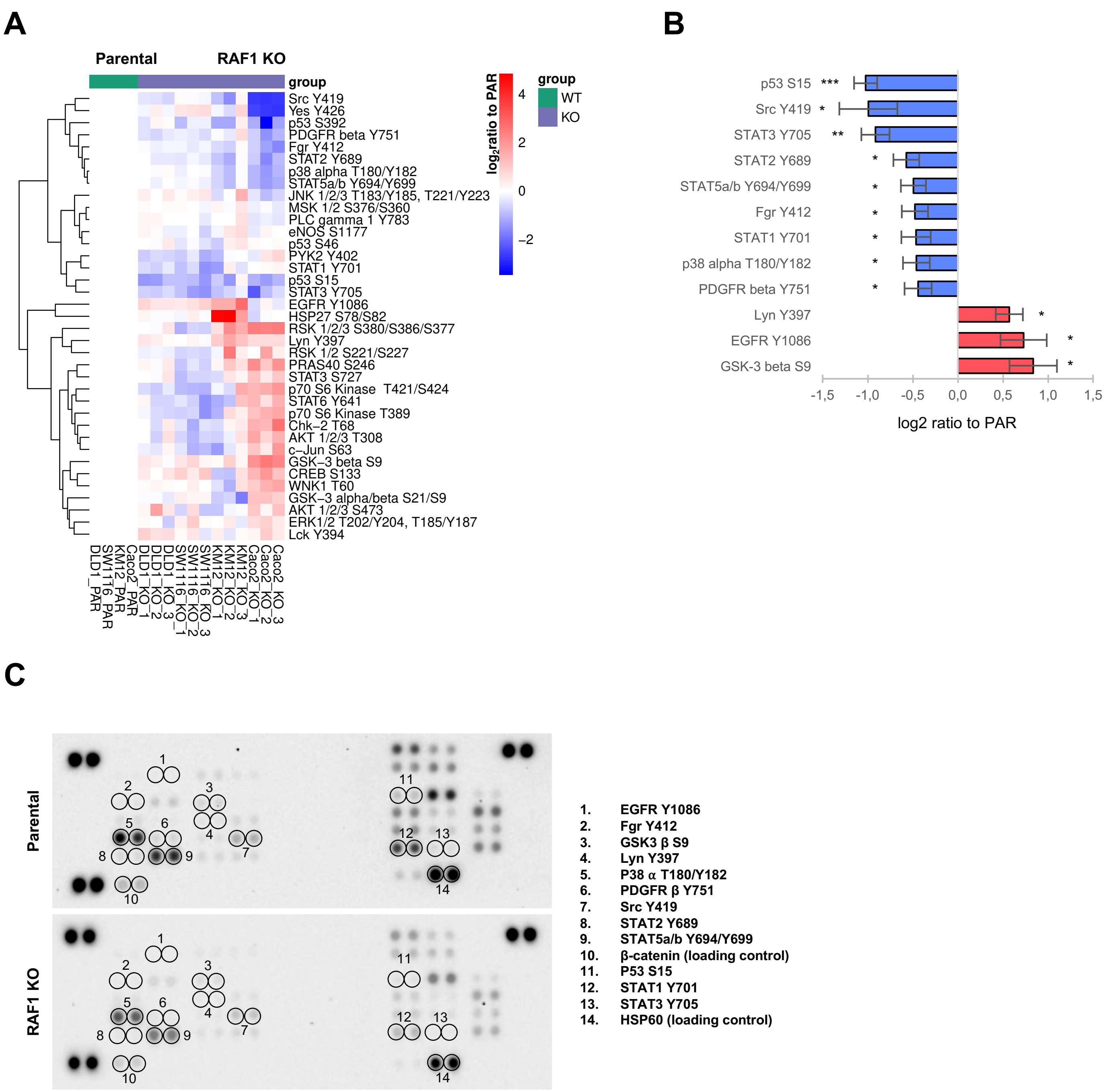

<u>**Supplementary Figure 6.** RAF1 phospho-kinase signature in human CRC cell lines</u>. **A**, 3D spheroids from cell lines were collected 5 days after spheroid formation and protein lysates were incubated on a proteome profiler™ array specific to phospho-kinases. Phosphoproteomic array analysis revealed increased and decreased phosphorylated proteins in absence of RAF1 for each cell line. **B**, Significantly deregulated phosphorylation sites common to RAF1 KO cells from DLD1, SW1116, KM12 and Caco2 when compared to the parental cell lines. Adjusted p-value at 0.05. **C**, Representative membranes of the human phospho-kinase proteome profiler™ array for SW1116 cell line, parental and RAF1 KO. Significantly deregulated phospho-kinases are indicated in the legend. β-catenin and HSP60 were used as loading controls. **B**, p*<0.05, p**<0.01, p***<0.001. n.s not significant

Supplementary Figure 6

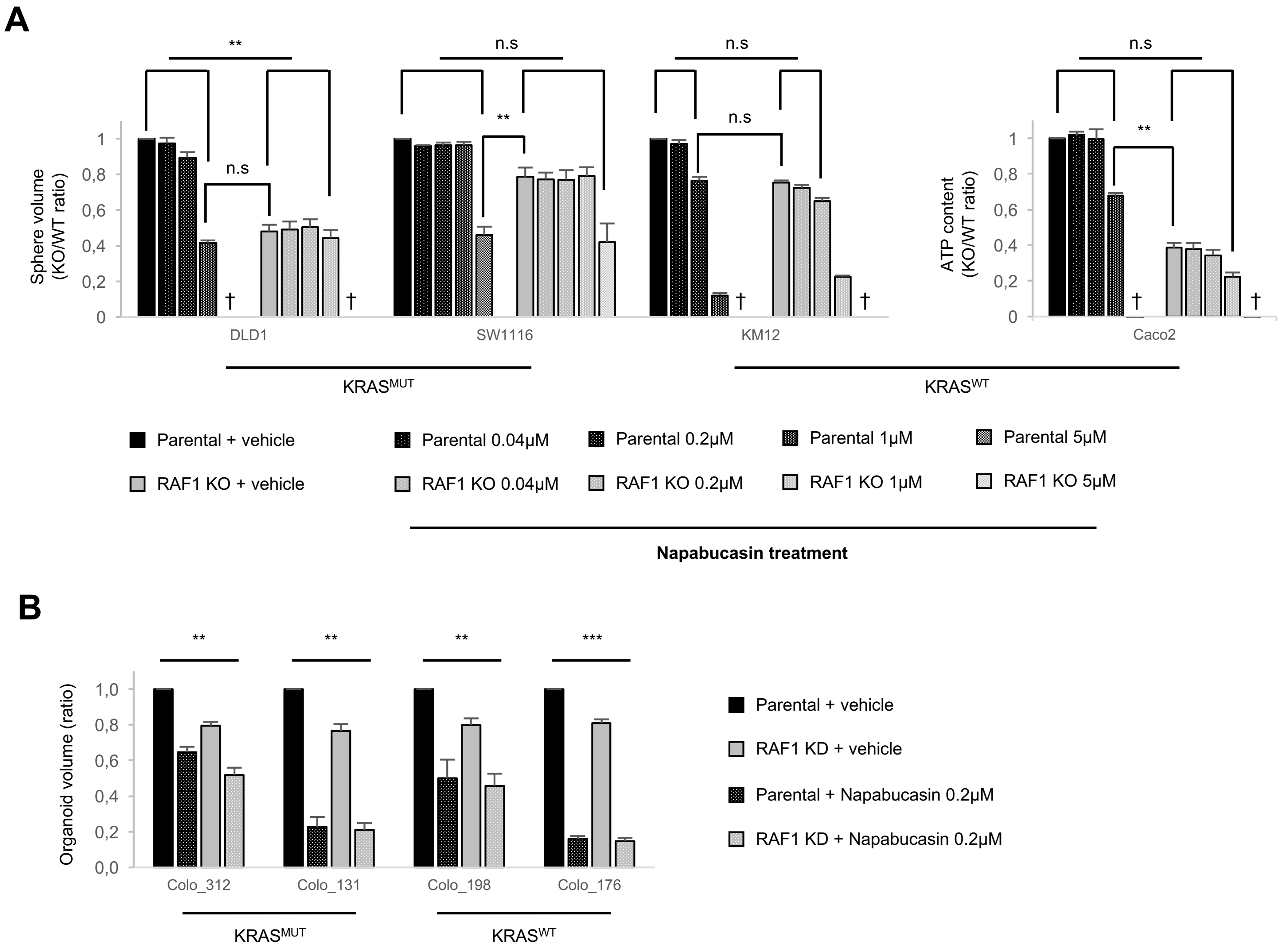

Supplementary Figure 7. Loss of RAF1 do not increase sensitivity to STAT3 inhibitor in human CRC cell lines and PDOs. **A**, Spheroids from parental and RAF1 KO CRC cell lines were treated for 5 days with either DMSO (vehicle), or four different concentrations of Napabucasin (0.04μM; 0.2μM, 1μM and 5μM). Spheroid volume or ATP content were determined at the end of the experiment, which was performed in triplicate. † lethal condition. **B**, Dox-induced/uninduced organoids expressing RAF1 shRNA were treated either with DMSO (vehicle) or Napabucasin (0.2μM). Volume of PDOs was measured after 8 days of treatment. Experiments were performed in triplicate. **A-B**, p*<0.05, p**<0.01, p***<0.001. n.s not significant

Supplementary Figure 7

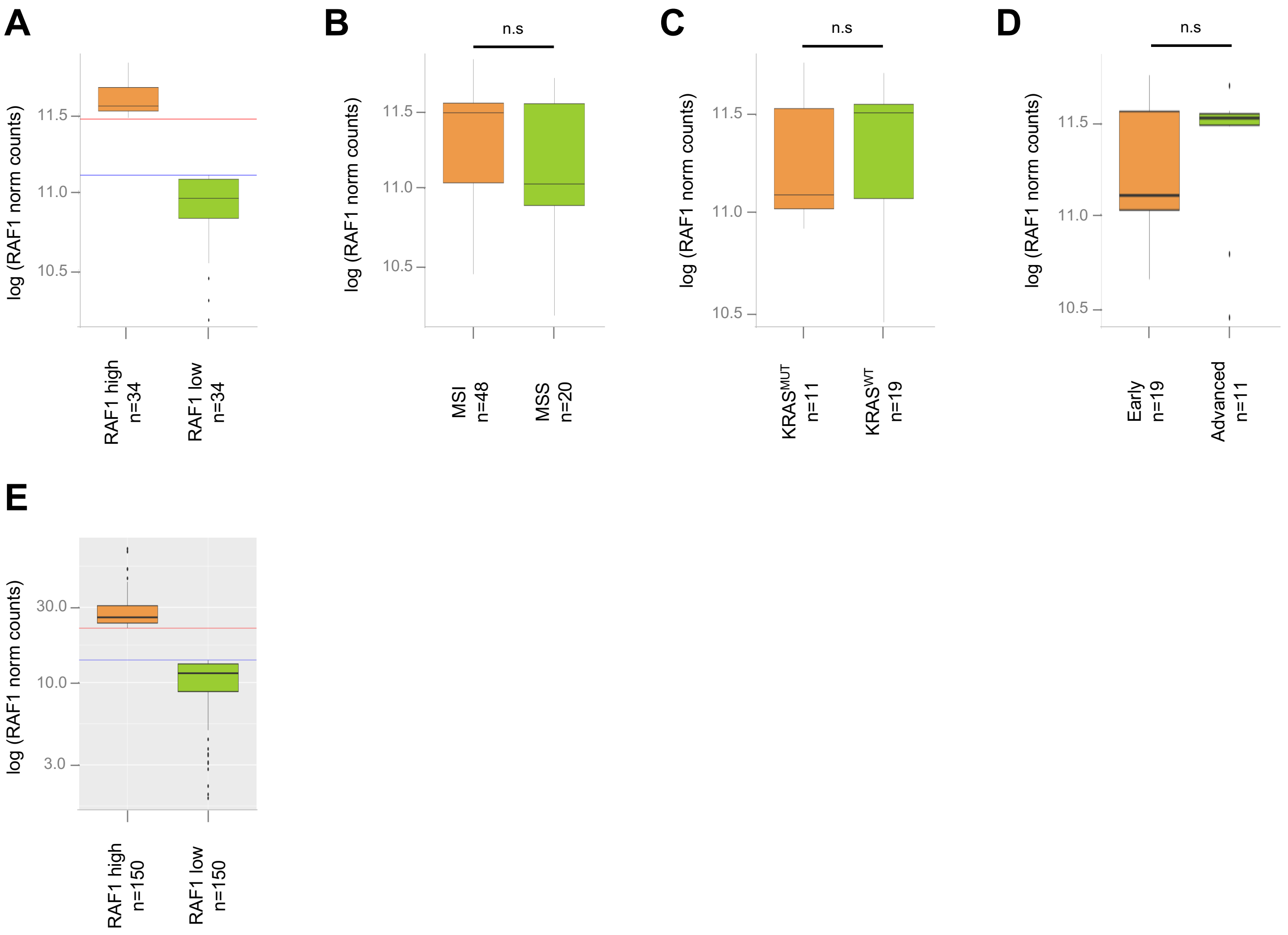

<u>**Supplementary Figure 8.**</u> <u>RAF1 expression does not correlate with KRAS or MSI/MSS status in CRC patients</u>. **A**, CRC primary tumors were segregated into quartiles according to RAF1 mRNA reads by RNAseq. **B**, Distribution of RAF1 expression in MSI and MSS tumors. **C**, Distribution of RAF1 expression in KRAS WT and mutated primary tumors within the MSI group. **D**, Distribution of RAF1 expression according to the tumor stage within the MSI group. **E**, LUAD primary tumors were segregated into quartiles according to RAF1 mRNA reads by RNAseq. **B-D**, n.s not significant

Supplementary Figure 8

	Gender		MS status		KRAS background		Tumor Stage		5-year Overall Survival (OS) [a]		5-year Relapse-free Survival (RFS) [a]	
	F	M	MSS	MSI	WT	MUT	Early (I-II)	Advanced (III-IV)	Alive	Dead	Yes	No
**RAF1 high (n=34)**	9	13	8	26	12	5	9	9	19	2	13	2
**RAF1 low (n=34)**	9	9	12	22	7	6	10	2	14	2	9	1
**Total**	**18**	**22**	**20**	**48**	**19**	**11**	**19**	**11**	**33**	**4**	**22**	**3**

Supplementary Table 4. Correlation between RAF1 expression and MS status, KRAS background and clinical parameters. RAF1 expression was determined by RNA sequencing. KRAS mutational status, stage of the disease, OS and RFS are known only for MSI patients. [a] 5-year OS and 5-year RFS were determined after diagnosis or resection. Due to the small number of patients, correlation between RAF1 expression levels and OS or RFS was not evaluated.

Supplementary Table 4

	Primer sequence 3' - 5'
ACTIN B forward	AGAGCTACGAGCTGCCTGAC
ACTIN B reverse	AGCACTGTGTTGGCGTACAG
BHLHE40 forward	CTGTTAGGACCCCACCCTTT
BHLHE40 reverse	AACCTCCCCAAACCACTACC
EMP1 forward	GACCTCATGCCATGGTCTTT
EMP1 reverse	CCCCTTGGATCCCATAGTTT
LDLR forward	ACCCCTACCCACTTCCATTC
LDLR reverse	ATCCCAACACACACGACAGA

Supplementary Table 5. List of the primers used in this study (qRT-PCR).

Supplementary Table 5
